# SARS-CoV-2 Spreads Globally Through the Object-to-Human Transmission of Cross-Border Logistics

**DOI:** 10.3389/fmicb.2022.918957

**Published:** 2022-06-23

**Authors:** Wenxia Shao, Qing Ye

**Affiliations:** ^1^Department of Clinical Laboratory, Affiliated Hangzhou First People’s Hospital, Zhejiang University School of Medicine, Hangzhou, China; ^2^Department of Clinical Laboratory, The Children’s Hospital, Zhejiang University School of Medicine, National Clinical Research Center for Child Health, National Children’s Regional Medical Center, Hangzhou, China

**Keywords:** SARS-CoV-2, COVID-19, transnational logistics, cold chain food, object to human, disinfection

## Abstract

With globalization, the demand for transnational logistics is growing rapidly. However, the object-to-human transmission of SARS-CoV-2 has been reported in transnational logistics production, transportation, storage, sales, and consumption. Every link of transnational logistics has the risk of spreading the COVID-19 pandemic. It is concluded that low temperatures, dry environments, and smooth surfaces are conducive to the long-term survival of SARS-CoV-2 on the surface of transnational goods. Epidemiological investigation and big data analysis show that the object-to-human transmission route of direct contact with contaminated cold chain goods plays a key role in the outbreak and transmission of the COVID-19 pandemic. This may be the most crucial reason for the global spread of SARS-CoV-2 caused by transnational logistics. It is an effective way to prevent the spread of SARS-CoV-2 from object-to-human through transnational logistics by strengthening the management of employees in all aspects of transnational logistics, carrying out comprehensive disinfection and quarantine of and guiding consumers to handle transnational goods properly.

## Introduction

COVID-19 is a public health emergency with the fastest transmission speed, the broadest range of infection, and the most challenging prevention and control in the past 100 years (Ye et al., 2020a,^2021^; Chen et al., 2021; Tian et al., 2021a,b; Han and Ye, 2022; Zhang et al., 2022), and it can cause multiple organ damage or even death (Tian and Ye, 2020; Ye et al., 2020a,b,c; Han and Ye, 2021; Han et al., 2021; Khodavirdipour et al., 2021a,b; Tian et al., 2021b). Vaccines and drugs are being developed (Khodavirdipour et al., 2020, 2021c,^2022^; Khodavirdipour, 2021). In the past, it was considered that the airborne transmission of SARS-CoV-2 was the dominant route of transmission (Jarvis, 2020). With globalization, the demand for transnational logistics is snowballing. It has been reported that some cold chain employees have been infected with SARS-CoV-2 due to exposure to contaminated imported cold chain products (Liu et al., 2020). In addition, live viruses are detected or isolated (Liu et al., 2020). It was found that SARS-CoV-2 could persist in contaminated frozen products. SARS-CoV-2 is also frequently detected in unopened packages and containers. Therefore, there is a risk of infection in contact with objects contaminated with SARS-CoV-2. Based on the above evidence, the World Health Organization has listed the transmission of SARS-CoV-2 from contaminated cold chain food to humans as a possible route of introduction and transmission. Thus, it has aroused people’s strong concern that cross-border goods will become the media of virus transmission between people and objects. However, it is unrealistic to stop international trade to prevent and control the epidemic situation for a long time. Therefore, cutting off the transmission route of SARS-CoV-2 from object to human has become a new global challenge. This paper analyzes the current situation and risk of epidemic spread brought by transnational logistics and puts forward some ideas on scientific and accurate prevention and control measures.

## Analysis of the Current Situation of SARS-CoV-2 Transmission Caused by Transnational Logistics

A complete logistics chain can be roughly divided into upstream production enterprises, transportation, storage, sales, and consumption. The contamination of goods in any chain link by SARS-CoV-2 may result in the infection of employees and consumers who contact these goods in its downstream links. In fact, several food-related enterprises around the world have clusters of outbreaks (Dyal et al., 2020; Middleton et al., 2020). The detection of live SARS-CoV-2 or viral nucleic acids in transnational logistics has also been reported from time to time (Jia et al., 2021; Wu et al., 2021). Even the staff of express delivery enterprises were infected with SARS-CoV-2 due to material transmission, which further caused the aggregation of human-to-human epidemics.

It is difficult for SARS-CoV-2 to proliferate and survive for a long time on the surface of dry objects at normal temperatures. However, the cold environment benefits the virus’s long-term survival (Holtmann et al., 2020). Direct contact with contaminated cold chain cargo may play a vital role in the outbreak and transmission of the COVID-19 pandemic. In September 2020, two cold chain workers were found to be asymptomatic for COVID-19 infection during routine nucleic acid testing in Qingdao. Subsequently, the Chinese Center for Disease Control and Prevention isolated the live virus from imported frozen cod packaging while tracing asymptomatic COVID-19 infection. The throat swabs of two workers and the surface swabs of frozen cod were isolated and sequenced. The results showed that the viruses were highly homologous (Liu et al., 2020). After that, SARS-CoV-2 was detected in frozen products and food packaging bags in many places (Beijing Xinfadi, Xinjiang Kashi, Shandong Qingdao, Liaoning Dalian). There are also risks in the storage of frozen food. In a recently reported case, a worker in a refrigerated warehouse in Tianjin, China, was infected with SARS-CoV-2. The environmental test results of the refrigerated warehouse showed that SARS-CoV-2 was positive on the outer package of frozen food and the door handle of the cold storage. These findings indicate the risk of object-to-human transmission of goods in refrigerated warehouses. In the sales link, a small-scale epidemic outbreak occurred in the Beijing Xinfadi market in June 2020. The traceability results show that the source of the virus is likely to be the cold chain imported food in the high incidence area of overseas epidemics (Pang et al., 2020). In addition, many other places in China also reported that SARS-CoV-2 nucleic acid was positive in imported cold chain food, internal and external packaging, transportation, and warehouses (Yu et al., 2021). The Chinese Center for Disease Control and Prevention isolated live SARS-CoV-2 from cold chain food packaging (Liu et al., 2020). The above evidence indicates that a low-temperature environment is conducive to the survival of viruses. The contamination of the surface of the object and its outer packaging may lead to the transmission of the virus to humans and then form a comprehensive transmission mode of humans. In addition, SARS-CoV-2 is also reported to be able to survive in aerosols for several hours, which may be due to the inclusion of a coating on its surface that provides some protection against external intrusion (Liu, 2004). Therefore, air transport is another important way to cross the international border.

Cross-border cold chain transportation may be a new method for SARS-CoV-2 transmission (Ceylan et al., 2020; Hu et al., 2021), which has aroused public concern and strong concern about the safety of cold chain food. However, the efficiency of SARS-CoV-2 transmission from contaminated surfaces to humans is unclear, and further research is needed (Ceylan et al., 2020).

**FIGURE 1:**
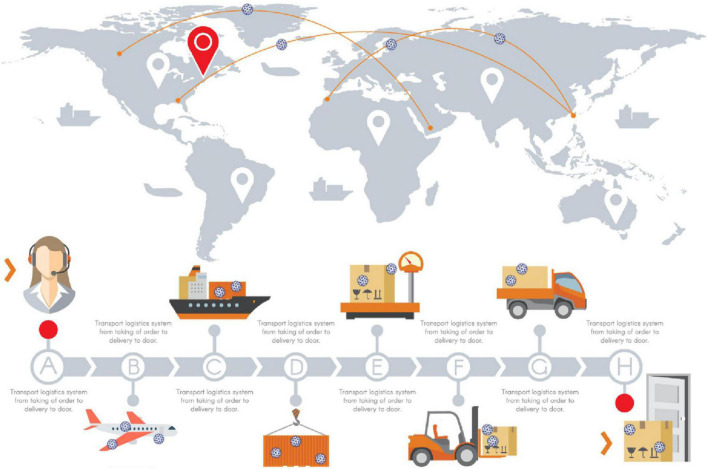
— SARS-CoV-2 spreads globally through cross-border logistics.

In summary, the risk of SARS-CoV-2 transmission exists in all links of transnational logistics (Figure 1). It may be that individuals with relatively weak immunity first infect SARS-CoV-2 from the surface of objects through contact and other means and then further cause human-to-human transmission.

## Risk Analysis of SARS-CoV-2 Transmission Caused by Transnational Logistics

It has been reported that SARS-CoV-2 has been detected on the surface of many objects. This indicates that SARS-CoV-2 has certain environmental persistence. The stay and survival of SARS-CoV-2 left on the object surface through the contact of infected patients is vital to cause the COVID-19 aggregation outbreak and spread. Therefore, the factors that affect the survival and residence of SARS-CoV-2 on the object surface determine the risk of transmission. The particle size of the virus is approximately a nanometer, and it is also a living body. The temperature and humidity of the environment affect the survival of the virus and affect its spread. Previous studies have confirmed that temperature is an essential factor in the transmission of SARS-CoV-2 since temperature changes directly affect the survival time of SARS-CoV-2 on the object surface (van Doremalen et al., 2013). In winter, a sharp outbreak of COVID-19 cases occurred in all countries in the Northern Hemisphere, adding new evidence for the relationship between SARS-CoV-2 and temperature (Holtmann et al., 2020). Bukhari et al. (2020) analyzed the weather patterns of the regions affected by COVID-19 worldwide. They found that 85% of the reported cases of COVID-19 occurred in areas with temperatures of 3–17^°^C and absolute humidity of 1–9 g/M^–3^. Only 15% of patients are reported in the tropics (Bukhari et al., 2020). Later, some Chinese scholars further found that when the temperature was lower than 3^°^C, the number of confirmed cases of COVID-19 was positively correlated with the temperature, and even warming could not alleviate the increase in the number of Xie and Zhu (2020).

Generally, with increasing temperature, the survival rate of the virus decreases (Tang, 2009). The reason is that temperature affects the activity of viral proteins and the stability of the viral genome, thus affecting the survival rate of the virus. The Australian team simulated the effect of temperature on the survival time of SARS-CoV-2 on the object’s surface in a P4 laboratory. When the humidity is controlled at 50% and 20^°^C, the virus on the object’s surface can survive for 28 days. The survival time at 40^°^C was less than 24 h (Riddell et al., 2020). The results also show that SARS-CoV-2 can survive on the surface of objects at room temperature. The longest survival time was even more than 9 days. SARS and MERS are similar to coronaviruses (Kampf et al., 2020a), and the survival time of SARS-CoV-2 can be reduced only at ambient temperature (Ceylan et al., 2020).

Environmental humidity is also an essential indicator of the spread speed and mortality of infectious diseases, especially the spread of the influenza virus. The transmission velocity and mortality rate were negatively correlated with humidity (Sooryanarain and Elankumaran, 2015). According to an epidemiological survey of COVID-19, humidity is indeed a factor affecting the mortality and transmission speed of SARS-CoV-2, especially when humidity is reduced in winter, and the fatality rate of COVID-19 will increase (Chen N. et al., 2020). When the relative temperature was 5.04–8.2^°^C, the daily confirmed cases decreased by 11–22%, while RH increased by 1% (Qi et al., 2020). In the future, the interaction mechanism needs to be further studied to clarify the specific effect of humidity on SARS-CoV-2 activity.

In addition, the stability of SARS-CoV-2 on object surfaces has been confirmed to be higher than that of other coronaviruses. At 22^°^C and 65% relative humidity, SARS-CoV-2 can survive for 4 days on glass and paper money, 7 days on smooth surfaces such as stainless steel and plastic, and 2 days on rough surfaces such as fabrics and wood (Dehbandi and Zazouli, 2020). Further study on the survival of viruses on stainless steel surfaces under different temperature and Rh conditions showed that SARS-CoV-2 inactivation was faster with increasing temperature and R (Casanova et al., 2010, see Table 1 for details).

**TABLE 1:** Survival time of SARS-COV-2 on different surfaces.

Object surface	Temperature (^°^C)	Relative humidity (%)	Survival time	References
Salmon	4		10d	Dai et al., 2020
	25		2d	Dai et al., 2020
Copper	21–23	40	4 h	van Doremalen et al., 2020
Copper	21–23	40	1d	van Doremalen et al., 2020
	Room temperature	65	4d	Chin et al., 2020
Glass	20–25	63	2d	Chan et al., 2020
	Room temperature	65	4d	Chin et al., 2020
Stainless steel	21–23	40	2d	van Doremalen et al., 2020
	Room temperature	65	7d	van Doremalen et al., 2020
Plastic	21–23	40	3d	van Doremalen et al., 2020
	Room temperature	65	7d	Chin et al., 2020

In conclusion, low temperature, a dry environment, and a smooth surface are high-risk factors for the global spread of COVID-19 caused by transnational logistics.

## Epidemic Prevention and Control Measures and Emergency Plan of Transnational Logistics

Given the risk of COVID-19 spreading through cold chain food, many authoritative scientific institutions in the world have put forward a series of prevention and control measures, such as being careful of humans and goods together, nucleic acid detection, and comprehensive preventive disinfection, to reduce the risk of epidemic transmission.

### Strengthen the Management of Transnational Logistics Practitioners

For first-line operators who are directly in contact with international goods, as well as other high-risk post personnel, record management should be implemented, and the posts should be relatively fixed to avoid cross operation with domestic express operators. All personnel in high-risk positions should receive epidemic prevention education and training and complete the COVID-19 vaccination. Closed-loop or closed management should be implemented for the staff in high-risk positions. A shift system with a certain work cycle is adopted. During the working period, centralized accommodation, closed management, point-to-point transfer between the workplace and the residence, and avoid contact with family members and the public. A careful record of personal action tracks should be made during the non-work period, and access to crowded places should be avoided. In handling international goods, first-line operators should strengthen personal health protection and wear protective equipment such as masks, gloves, goggles, etc., during the whole process and perform temperature detection before going on and off duty. For logistics support personnel such as cleaners, outsourcing transport vehicle drivers, and other relevant personnel, we should also carry out risk screening and prevention and control of imported epidemics to avoid cross-infection of viruses.

### Strengthen the Management of Transnational Goods

Imported cold chain food production should be traceable. Epidemic screening, sample collection, nucleic acid testing, customs clearance, and environmental disinfection should be carried out. After entering the production process, the goods must be thoroughly disinfected immediately. Disinfection of the surface of cross-border goods is the key to inhibiting the spread of SARS-CoV-2 in the environment (Carraturo et al., 2020; Kampf et al., 2020a). At present, liquid chemical disinfectants are the most commonly used method to disinfect the surface of objects (Russell, 2003; Goyal et al., 2014; Dindarloo et al., 2020; Wang et al., 2020). For contact transmission caused by packaging, chemical disinfectants can greatly reduce the risk of transmission (Godoy et al., 2021). However, unlike traditional disinfection, the disinfection of cold chain goods is a special operation at low temperatures. Traditional chemical disinfectants are generally practical only at room temperature. In cold chain logistics, once the traditional chemical disinfectant is sprayed on the container, it is easy to freeze, so it is difficult to disinfect effectively. In addition, using traditional liquid disinfectants to disinfect cargo is time-consuming and tedious to perform manually. In addition, too much inventory also easily causes disinfection blind area problems. Ozone and other gas disinfectants are substitutes with a high degree of automation, no blind area in disinfection, and sound effects of low-temperature disinfection (Blanco et al., 2021; Criscuolo et al., 2021; Tizaoui et al., 2022). Ozone disinfection technology has achieved satisfactory results in the actual disinfection operation of cold chain logistics.

However, especially when using traditional chemical disinfectants, special attention should be given to possible secondary pollution in the disinfection process, including the risk of contamination of drinking water, the accumulation of toxic substances, and environmental pollution. Therefore, in the standardized disinfection stage of cold chain goods, it is suggested to popularize the application of the green disinfection method and study how to improve its performance and economic benefits.

Logistics-related infrastructure improvement: Cross-border logistics, especially frozen fresh food and frozen seafood, are basically small pieces that are directly in contact with people from packaging, transportation, and storage, which virtually increases the risk of workers being exposed to viruses and spreading viruses. In the face of the epidemic, it is necessary to strengthen the automation level of the above links. The possibility of SARS-CoV-2 contamination of cargo surfaces can be reduced through automated and standardized packaging equipment. Exposure to transport links can be reduced through automated loading and unloading vehicles and enclosed transport. The traditional port automation transformation achieves remote operation to achieve “zero contact.”

### Guide Consumers to Handle Transnational Goods Properly

For the outer package of cold chain goods (Khokhar et al., 2020; Sharafi et al., 2021) or ultraviolet disinfection device (Miller et al., 2013; Malhotra et al., 2020; Fadaei, 2021; Gidari et al., 2021) can be used for disinfection. Consumers should wash their hands after unpacking express packages. While unpacking the containers, do not touch your mouth, eyes, or nose with unclean hands. High-temperature sterilization is a common sterilization method. Heat accumulation on the virus’s surface can rapidly lead to protein denaturation of the virus envelope, thus playing a role in killing the virus (Kampf et al., 2020b). Therefore, high-temperature disinfection can also produce a pronounced killing effect on SARS-CoV-2 (Chen H. et al., 2020). Therefore, consumers are advised to cook seafood and other food before eating it to minimize the possibility of SARS-CoV-2 transmission. For vegetables and fruits that need to be cleaned, the new ozone microbubble water technology can be used to achieve efficient cleaning and disinfection at the same time.

## The Unavoidable Hurdles/Limitations in Preventing Surface-Contact Transmission of SARS-CoV-2

Although various measures have been taken to block the transmission of surface contact of SARS-CoV-2, and these measures have achieved remarkable results, this risk cannot be completely eliminated. We have carried out strict inspections and quarantine on imported goods, but it is difficult for us to sample the inside of the package, and there is a certain risk of missing the sample inspection. We can carry out strict disinfection on the surface of the food and consider food safety, and we cannot have chemical disinfectants to disinfect the food itself. Although we can improve the level of automation in the whole logistics process, people will always be indispensable, and it is impossible to avoid human contact with goods completely.

## Conclusion

Every link of transnational logistics risks spreading the COVID-19 epidemic through the object to humans, especially cold chain food. Strengthening the management of employees in all aspects of transnational logistics, carrying out comprehensive disinfection and quarantine of transnational goods, and properly handling transnational goods by consumers are effective means to prevent the transmission of SARS-CoV-2 from goods to people through transnational logistics.

## Author Contributions

QY led the manuscript writing. WS developed the initial concept and framework for the manuscript and oversaw the drafting. Both authors contributed to the content, drafting, and critical review of the manuscript.

## Conflict of Interest

The authors declare that the research was conducted in the absence of any commercial or financial relationships that could be construed as a potential conflict of interest.

## Publisher’s Note

All claims expressed in this article are solely those of the authors and do not necessarily represent those of their affiliated organizations, or those of the publisher, the editors and the reviewers. Any product that may be evaluated in this article, or claim that may be made by its manufacturer, is not guaranteed or endorsed by the publisher.
